# Engineering and characterising a novel, highly potent bispecific antibody iMab-CAP256 that targets HIV-1

**DOI:** 10.1186/s12977-019-0493-y

**Published:** 2019-11-08

**Authors:** Tumelo Moshoette, Stuart Alvaro Ali, Maria Antonia Papathanasopoulos, Mark Andrew Killick

**Affiliations:** HIV Pathogenesis Research Unit, Department of Molecular Medicine and Haematology, Faculty of Health Sciences, University of the Witwatersrand, 7 York Road, Parktown, Johannesburg, 2193 South Africa

**Keywords:** HIV-1 prevention, HIV-1 therapy, Broadly neutralizing antibodies, Bispecific antibodies

## Abstract

The existing repertoire of HIV-1 patient derived broadly neutralising antibodies (bNAbs) that target the HIV-1 envelope glycoprotein (Env) present numerous and exciting opportunities for immune-based therapeutic and preventative strategies against HIV-1. Combination antibody therapy is required to ensure greater neutralization coverage and limit Env mediated escape mutations following treatment pressure. Engineered bispecific bNAbs (bibNAbs) assimilate the advantages of combination therapy into a single antibody molecule with several configurations reporting potency enhancement as a result of the increased avidity and simultaneous engagement of targeted epitopes. We report the engineering of a novel bibNAb (iMab-CAP256) comprising the highly potent, CAP256.VRC26.25 bNAb with anticipated extension in neutralization coverage through pairing with the host directed, anti-CD4 antibody, ibalizumab (iMab). Recombinant expression of parental monoclonal antibodies and the iMab-CAP256 bibNAb was performed in HEK293T (Human embryonic kidney 293 T antigen) cells, purified to homogeneity by Protein-A affinity chromatography followed by size exclusion chromatography. Antibody assembly and binding functionality of Fab moieties was confirmed by SDS-PAGE (sodium dodecyl sulphate polyacrylamide gel electrophoresis) and ELISA, respectively. Breadth and potency were evaluated against a geographical diverse HIV-1 pseudovirus panel (n = 20). Overall, iMab-CAP256 demonstrated an expanded neutralizing coverage, neutralizing single, parental antibody resistant pseudovirus strains and an enhanced neutralization potency against all dual sensitive strains (average fold increase over the more potent parental antibody of 11.4 (range 2 to 31.8). Potency enhancement was not observed for the parental antibody combination treatment (iMab + CAP256) suggesting the presence of a synergistic relationship between the CAP256 and iMab paratope combination in this bibNAb configuration. In addition, iMab-CAP256 bibNAbs exhibited comparable efficacy to other bibNAbs PG9-iMab and 10E08-iMab previously reported in the literature. The enhanced neutralization coverage and potency of iMAb-CAP256 over the parental bNAbs should facilitate superior clinical performance as a therapeutic or preventative strategy against HIV-1.

## Introduction

HIV-1 patient-derived broadly neutralising antibodies (bNAbs) develop in approximately 20% of infected patients [1]. The most potent and broadly neutralizing of these show promise in novel HIV-1 prevention and therapeutic strategies. Characterization of these bNAbs has identified five conserved regions of vulnerability on the HIV-1 envelope glycoprotein (Env) spike. These include the membrane-proximal external region of gp41 e.g. 10E8 [2], the CD4 binding site e.g. VRC01 and N6 [3, 4], the apical V1–V2 glycan supersite (e.g. PG9, CAP256-VRC26.25), the V3 glycan (e.g. PGT128) on the outer domain of gp120 [5–7] and the N332 glycan supersite (e.g. 2G12, PGT121, PGT128 and PGT135 [8]). More recently a quaternary, discontinuous epitope spanning the gp120-gp41 interface has been identified, targeted by PGT151, 3BC315 and 35022 [9, 10]. Additionally, non-patient derived antibodies targeting the HIV-1 primary or co-receptors on host target cells, display extraordinary neutralization breadth and potency against diverse viral isolates given their obligatory requirement in viral entry. Ibalizumab, a humanized, anti-CD4 antibody binds the second domain of human CD4 and prevents HIV-1 infection in a non-competitive manner [11–13], whereas PRO140 competitively inhibits HIV-1 entry through binding the extracellular loop 2 region of the chemokine receptor 5 [14].

As a preventative strategy, bNAbs have clearly demonstrated utility in both humanised—mouse and rhesus macaque challenge models. Passive antibody transfer studies have shown to protect against intravenous [15, 16] or mucosal challenge routes [intravaginal [17–20], intrarectal [21–25] and oral [26, 27] (MTCT infant macaque challenge model)]. Encouraged by these results, a major clinical trial (HVTN 703/HPTN 081 and HVTN 704/HPTN 085) is underway to evaluate the ability of bNAb VRC01 to protect against HIV-1 acquisition in humans and to establish the bNAb concentration required for protection [28]. In addition, vector mediated immunoprophylaxis may propel such strategies into mainstream, human application. Long-lived in vivo expression of bNAbs has been shown to confer protection against intravenous and mucosal challenge routes in both the humanised mouse and rhesus macaque challenge models [29–31].

As a therapy, bNAbs offer several advantages over existing antiretroviral drug therapies (ART), including; lower toxicity, improved pharmacokinetics [32], Fc-mediated immune effector function [33–35] and extension of treatment options to patients failing conventional ARTs. However, bNAb monotherapy may be limited due to rapid selection of viral escape mutations leading to viremia rebound [36–39]. This may be circumvented with the use of combination bNAb treatment, extending long-term viral suppression and reducing the emergence of viral escape [40–42].

Pertinent to both these strategies is the associated breadth and potency of the selected bNAbs. In the context of prevention, breadth of selected bNAbs must display adequate coverage to protect against a phylogenetically diverse viral swarm challenge [21] and be present in sufficient concentrations at the site of transmission in order to be effective. This may be subject to HIV-1 and host population bias including: the predominant circulating subtype and founder virus, HIV-1 transmission routes, host genetics, etc. Whilst in a therapeutic setting, broader coverage may be required to combat HIV-1 evolution under the selective pressure of the bNAb action. To date, no single bNAb achieves 100% neutralization against the available HIV-1 multi-subtype, Env-pseudotyped virus panels routinely used in the laboratory to represent clinically relevant HIV-1. Potency of bNAbs is also a critical determinant in protection, as lower mean plasma levels of the more potent PGT126 bNAbs demonstrate superior protection when compared to the less potent b12 bNAb in a humanized mouse model [43]. Overall, neutralization breadth and potency against selected HIV-1 pseudovirus panels can be improved with a combination of bNAbs [44, 45]. However, when translated into a clinical setting, the need to produce two or three different antibodies brings additional costs and regulatory considerations, highlighting the need for a better way of incorporating bNAbs as preventative agents [46].

Engineered bispecific (bi-) and trispecific bNAbs (tribNAbs) present a creative alternative to bNAb combination therapy. These antibody configurations comprise dual [47–51] or multi [52, 53] epitope targeting capabilities in a single antibody molecule. Paratope combinations include those that may solely target the HIV-1 Env [50, 52, 53] or combined Env and the relevant host-cell receptor targeting modalities [47–49, 51, 54]. BibNAbs have demonstrated exceptional improvement in neutralization coverage and potency relative to their parental antibodies [49, 50]. In particular, the 10E08-PRO140 and 10E08-iMab bibNAbs achieved 99% and 100% neutralization coverage of a 118 pseudovirus panel, respectively [49].

HIV-1 subtype C continues to dominate the global pandemic, accounting for ~ 47% human infections. Unsurprisingly, the geographical distribution of subtype C strains overlap with those regions most severely affected by the HIV-1 pandemic, including Eastern and Southern Africa. The CAP256.VRC26.25 bNAb was isolated from an HIV-1 subtype C patient and targets a quaternary, glycan dependent epitope of the V1/V2 apical loop of the HIV-1 Env spike [7, 55]. CAP256.VRC26.25 displays exquisite potency against sensitive viral isolates (median 50% inhibitory concentration (IC_50_) of 0.001 µg/ml) yet reduced neutralization breadth, achieving 57% neutralization of diverse subtypes and 70% of subtype C isolates [55]. Julg and colleagues [23] demonstrated the superior in vitro potency of CAP256-VRC26.25-LS (LS-incorporation of the LS mutation into the Fc portion to increase in vivo antibody half-life) mediated complete protection at previously unreported, low serum levels (< 0.75 µg/ml) against intrarectal, SHIV challenge in rhesus macaques. Furthermore, in 2016, Wagh and colleagues [45] reported on the optimal bNAb combinations for the treatment and prevention of HIV-1 subtype C infection. The authors concluded that the best-in-class 2, 3 and 4 bNAbs combinations all contained CAP256-VRC26.25, with preference for the 3 or 4 combinations due to overall coverage and maximal percent inhibition. Taken together, these studies highlight the potential clinical benefit that may be derived from CAP256.VRC26.25, with the possibility of specific tailoring to the treatment and/or prevention of subtype C infections. However, its usage may ultimately be confined to combination immunotherapy due to limited neutralization breadth. To this end, we sought to improve the breadth of CAP256.VRC26.25 through engineering a novel bibNAb (iMab-CAP256) and pairing it with the host cell directed anti-CD4 antibody, ibalizumab (iMab). IMab was selected as it demonstrates an expanded coverage over CAP256.VRC26.25, neutralizing > 90% of diverse HIV-1 Env subtypes at a median IC_50_ < 0.1 µg/ml. In addition, iMab has an established safety record in human clinical trials [56–58], receiving FDA (Food and Drug Administration) approval for treatment of multidrug resistant HIV-1 [59] and it exhibits potency and breadth enhancement in bibNAb conformations [47, 49]. Below we describe the engineering of iMab-CAP256 bibNAb, confirm the functionality of individual Fab binding regions in the bispecific antibody configuration and demonstrate an improved breadth and potency relative to the parental monoclonal antibodies or combination treatment, against selected HIV-1 pseudoviruses.

## Methodology

### Reagents used in this study

The single plasmid, mAb expression vector (pMin) was generously donated by Balazs [30, 31] and modified to remove the CMV promoter and ITR flanking regions for optimal in vitro antibody expression. The following reagents were obtained from the NIH AIDS Research and Reference Reagent Program, Division of AIDS, NIAID (contributor in parentheses): Antibody expression vectors (heavy and light chain) for the 10E8 mAb (Dr. Mark Connors [2]) and VRC01 mAb (Dr. John Mascola [3]). The PG9-ibalizumab (PG9-iMab) bispecific antibody (Dr. David Ho and Dr. Craig Pace [47]).

Reagents for HIV-1 pseudovirus production including: TZM-bl (JC53-bl) reporter cell line (Drs. John C. Kappes, Xiaoyun Wu and Transzyme Inc. [60–64]), pSG3^Δenv^ (Drs. John C. Kappes and Xiaoyun Wu [64, 65]) and 20 complementing HIV-1 Env plasmids 25710, 246-F3.C10.2, 398f1 and CH119 (Drs. C. Williamson, M. Hoelscher, L. Maboko, and D. Montefiori [66, 67]), CNE8 and CNE55 (Drs. L. Zhang, H. Shang and D. Montefiori [66, 68]), PVO.4 and QH0692.42 (Drs. D. Montefiori, F. Gao and M. Li [69]), CAP210.2.00.E8 and CAP45.2.00.G3 (Drs. L. Morris, K. Mlisana and D. Montefiori [70]), DU156.12 (Drs. D. Montefiori F. Gao, S. Abdool Karim and G. Ramjee [70, 71]), DU422.01 (Drs. D. Montefiori, F. Gao, C. Williamson and S. Abdool Karim [70, 71]), ZM53 M.PB12 and ZM135 M.PL10a (Drs. E. Hunter and C. Derdeyn [70]), X1632 (Dr. D. Montefiori [66, 72]), TRO11 (Drs. F. Gao and D. Montefiori [66, 69]), X2278 and BJOX2000 (Drs. M. Thomson, A. Revilla, E. Delgado, and D. Montefiori [66]), CE0217 and CE1176 (Drs. R. Swanstrom, L. Ping, J. Anderson, and D. Montefiori [66]). The HIV-1 global pseudovirus panel is included in the abovementioned 20 plasmids.

### Bispecific antibody construct design and synthesis

**Fig. 1:**
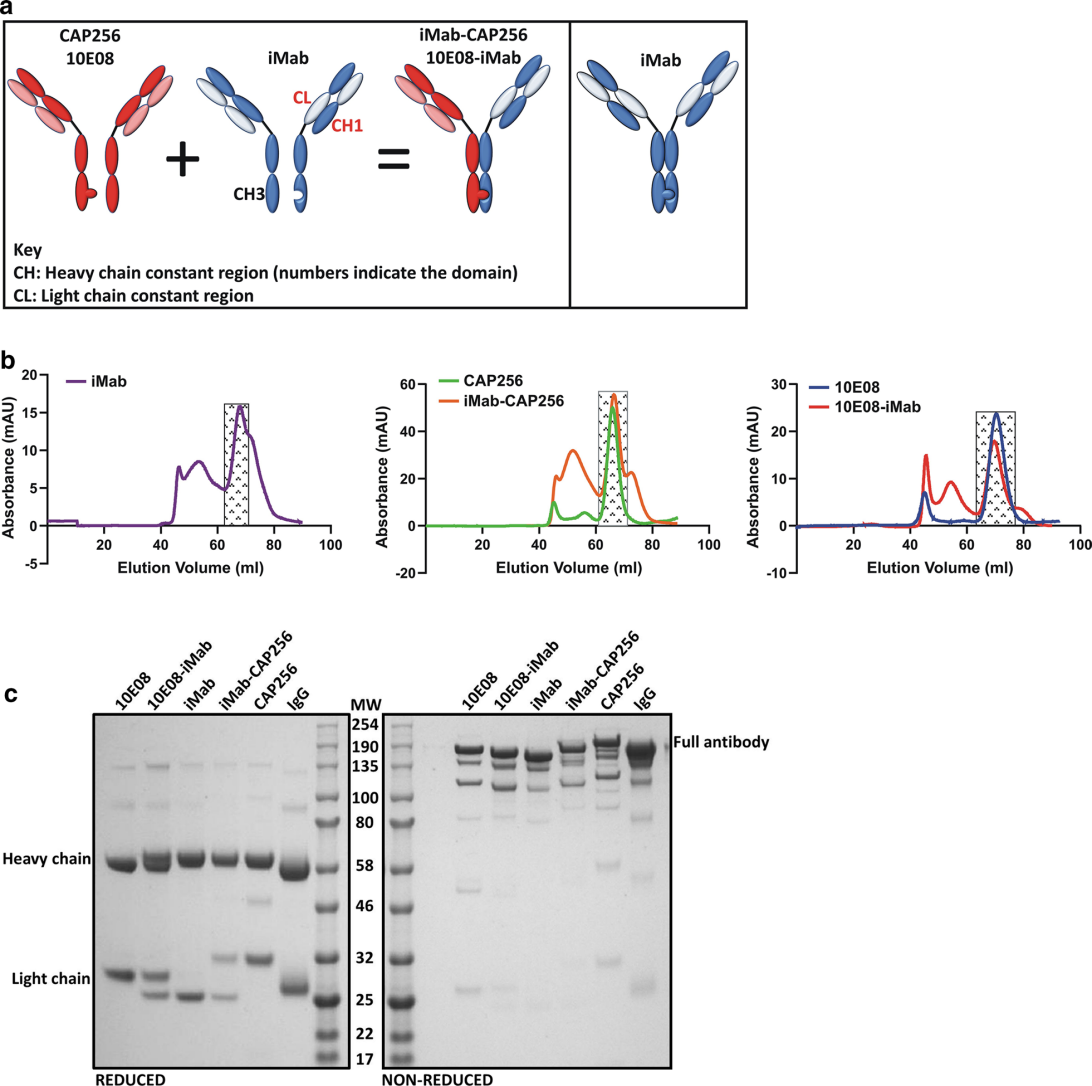
Bispecific antibody design, purification and SDS-PAGE analysis. **a** Left panel: Schematic of the iMab-CAP256 or 10E08-iMab bibNAb, with ‘knob’ mutations introduced into the CH3 region of CAP256 or 10E08 and complementing ‘hole’ mutations introduced into the CH3 region of iMab. Additionally, the CH1 region and the CL region of iMab were interchanged to create a bibNAb with a Crossmab^CH1-CL^ configuration. **a** Right panel: Schematic of iMab engineering using knob-in-a-hole and CrossMab^CH1-CL^ to serve as a suitable assembly control. **b** Size exclusion chromatograms of Protein-A purified antibodies. Shaded rectangles represent collected and pooled fractions corresponding to the monomeric antibody conformation. **c** SDS-PAGE analysis of antibodies under reducing and non-reducing conditions post size exclusion chromatography. Purified human IgG was included as a positive control. Molecular weight (MW) is indicated in kDa

Bispecific antibody constructs were generated according to previously published protocols, and include the knob-in-a-hole [50] and CrossMab^CH1-CL^ [73] technologies, to ensure correct in vitro assembly of bispecific antibodies. The corresponding iMab [12], CAP256 [55] and 10E08 [2] fragment antigen-binding (Fab) regions (PDB: 3O2D, 5DT1 and 4G6F respectively) were assembled in silico into the bispecific antibody conformation using SnapGene. The CrossMab^CH1-CL^ mutation were added to iMab along with the “hole” mutations; T366S, L368A, Y349C and Y407V [50, 73] (Fig. 1a). The complimenting “Knob” mutations, T366W and S254C were introduced into the HIV-1 Env, targeting bNAbs, CAP256 and 10E08 (left panel, Fig. 1a). The modified amino acid sequences were sent to GeneArt for codon optimisation and gene synthesis. Unmodified amino acid sequences to express the parental monoclonal control antibodies were generated in tandem (CAP256 and 10E08). The iMab parental antibody contained the respective knob-in-a-hole and CrossMab^CH1-CL^ to serve as a suitable assembly control (right panel, Fig. 1a). Synthesized antibody gene constructs were sub-cloned into a mammalian expression vector provided courtesy of Balazs [30, 31]. Large scale plasmid production was performed in *E. coli* DH5α cells and purified using the Qiagen Maxi plasmid isolation kit.

### Antibody expression and purification

Antibodies were transiently expressed in the HEK293T cell line using a 1:3 DNA to PEI (Polysciences Inc.) transfection protocol. Twenty-four hours post transfection, the cell culture media was discarded and replaced with serum free media, SFMII (Thermo Fisher Scientific) supplemented with 2 mM glutamax (Thermo Fisher Scientific). Cell culture supernatants containing the expressed antibodies were harvested and replaced every 48 h for a maximum of 10 days. Harvested culture supernatants were centrifuged and filtered. Antibodies were purified by Protein-A affinity chromatography (Sigma-Aldrich) followed by size exclusion chromatography (SEC) (Fig. 1b) on a Superdex 200 PG 16/600 HiLoad column (GE Healthcare) to remove protein aggregates and oligomeric forms. Collected fractions were concentrated using an Amicon centrifugal-filtration device with a molecular weight cut-off 50 kDa. The purified proteins were resolved on an SDS-PAGE gel to confirm their purity.

### Protein purification

HIV-1 monomeric (gp120_FVC_M) or trimeric (gp140_FVC_GCN4 or gp140_FVC_SOSIP) Env conformations were recombinantly expressed by transient transfection (using PEI) or stably transfected 293F cell lines [74]. All three constructs comprise the inferred Founder virus consensus C sequence (FVC) described previously [75]. The gp120_FVC_Monomer and gp140_FVC_GCN4 constructs were available in our laboratory and have been described previously [74]. The gp140_FVC_SOSIP trimer was engineered using specific amino acid substitutions as described previously [76] (HxB_2_ numbering system): A501C-T605C (gp120-gp41 intra-subunit disulphide bond), I559P (trimer stabilization), enhanced furin cleavage site by replacing REKR with RRRRRR in gp120 and introduction of a stop codon at position 664 to truncate the gp41_ECTO_ domain, increasing solubility and homogeneity. The gp140_FVC_SOSIP were exclusively expressed by transient co-transfection with a furin encoding plasmid (1:4 ratio of furin to gp140_FVC_SOSIP) in HEK293T cells. Twenty-four hours post transfection; culture media was replaced with Freestyle 293 Expression media (Thermo Fisher Scientific). Harvested culture media, containing the expressed Env, was centrifuged and filtered to remove cellular debris and purified using *G. nivalis* lectin affinity chromatography, and SEC, as described previously [74]. Recombinant human two-domain CD4 (2dCD4) was expressed in *E. coli* BL21 (DE3) and purified from inclusion bodies, under denaturing conditions using IMAC, and refolded in a series of glutathione containing phosphate buffers, as described previously [77].

### Functional evaluation of bibNAb Fab moieties

The functionality of the Fab moieties was determined by an ELISA. To determine HIV-1 Env binding, decreasing concentrations of HIV-1 Env (gp140_FVC_GCN4, gp120_FVC_Monomer or gp140_FVC_SOSIP) were captured by immobilized *G. nivalis* lectin (Sigma) (200 ng/well) on a 96 well ELISA plate (Maxisorb—Nunc). To assess binding to human CD4, decreasing concentrations of bacterially expressed 2dCD4 was immobilized directly on the 96 well ELISA plate. Plates were blocked for an hour [Dulbecco’s phosphate buffered saline (Sigma), 0.05% (v/v) Tween 20 and 1% BSA]. Binding of bibNAbs and parental antibodies was then performed at a concentration of 1 µg/ml, with the exception of VRC01 which was used at a final concentration of 0.5 µg/ml. Bound antibodies were then detected using anti-human, horseradish peroxidase-linked secondary antibody (GE Healthcare) and standard chromogenic methodologies. Absorbance was read at 450 nm using an iMark microplate reader (Bio-Rad). For this assay, gp120_FVC_Monomer was used as a negative control.

### HIV-1 in vitro neutralisation assay

Antibody mediated neutralisation was assessed against a panel of 20 geographically diverse pseudoviruses using a single round HIV-1 pseudovirus infectivity assay in the TZM-bl reporter cell line, as described previously [78]. Briefly, antibody concentrations were prepared by tenfold serial dilution and tested at a maximum, final concentration of 4 µg/ml (10E08, 10E08-iMab and PG9-iMab) or 1 µg/ml (CAP256, iMab-CAP256 and iMab) or 0.5 µg/ml per parental antibody for parental combination treatments (total IgG concentration 1 µg/ml). Two hundred TCID_50_ (50% Tissue Culture Infectious Dose) of pseudovirus was added to each well and incubated for an hour at 37 °C in 5% CO_2_. Following this, 100 µl of TZM-bl cells [1 × 10^5^ cells/ml in complete DMEM supplemented with DEAE Dextran (Sigma)] were added to each well and the plate was incubated for 48 h at 37 °C in 5% CO_2_. The final concentration of DEAE Dextran in the assay was 20 µg/ml. Luciferase was determined using Bright Glo Luciferase reagent (Promega) according to the manufacturer’s instructions and quantified using the Promega Glomax Explorer luminometer (Promega). Cell only and virus only control wells were included as appropriate controls. IC_50_ values were calculated using the non-linear regression function in GraphPad Prism 7 and represent the antibody concentration (µg/ml) required to achieve 50% pseudovirus inhibition.

### Statistical analysis

Comparisons of median neutralization IC_50s_ between the iMab-CAP256 bibNAb and the parental antibodies (CAP256 or iMab) or parental antibody combination (iMab + CAP256) or additional bibNAbs (10E08-iMab or PG9-iMab) were performed using a non-parametric, *t*-test (Wilcoxon matched-pairs signed rank test) and two-tailed *p*-value. Where multiple comparisons between groups were performed, the level of significance was adjusted using Bonferroni correction (α/the number of comparisons). *p*-values were considered significant when the *p*-value was < 0.0166 using the Bonferroni correction for 2 × 2 comparison of the iMab-CAP256 bibNAb to each parental antibody (iMab or CAP256) or the parental antibody combination (iMab + CAP256) or when a *p*-value was < 0.025 for 2 × 2 comparison of iMab-CAP256 with each bibNAbs, 10E08-iMab or PG9-iMab. Statistical analyses were performed using GraphPad Prism 7.

## Results

### Antibody design and expression

iMab-CAP256 was based on the design of the 10E08-iMab bibNAb described by Huang et al. [49] and was engineered using CrossMab^CH1-CL^ [73] and knob-in-a-hole [50] mutations (left panel, Fig. 1a). The introduction of these mutations has previously been shown not to affect the antigen binding regions, yet allows for preferential heterologous heavy and light chain assembly, resulting in the production of functional bispecific antibodies in vitro [50, 73]. Constructs for the expression of 10E08-iMab, as described by Huang et al. [49] were synthesised in parallel and served as an appropriate comparative control for functional assembly, bispecificity and enhanced HIV-1 neutralisation. Sufficient quantities of bibNAbs (iMab-CAP256 and 10E08-iMab) and the parental monoclonal antibodies (CAP256, iMab and 10E08) were expressed in HEK293T cells and purified using Protein-A agarose (Fig. 1b). The SEC chromatograms indicate conformational heterogeneity of the Protein-A purified antibodies particularly for those antibodies expressed from the pMin backbone construct (iMab, CAP256, iMab-CAP256 and 10E08-iMab) compared to the parental 10E08 antibody construct (Fig. 1b). Elution fractions corresponding to the anticipated, correctly assembled antibody conformation (peak retention volume approximately 65 ml) were collected and pooled (shaded boxes, Fig. 1b). Following SEC purification, the antibodies were resolved on an SDS-PAGE under reduced and non-reduced conditions to confirm structural integrity and degree of purity (Fig. 1c). Under reducing conditions, both heavy and light chain resolved at the anticipated molecular weights across all antibodies (Fig. 1c). Two distinct light chain bands were discernible for both bibNAbs (iMab-CAP256 and 10E08-iMab) and correspond to the respective parental monoclonal antibody light chain constituents (left gel, Fig. 1c). Similarly, a discernible separation of the 10E08-iMab bibNAb heavy chains was observed and accurately corresponded to a slight difference in the migration of the iMab and 10E08 parental heavy chains respectively (left gel, Fig. 1c). Under non-reducing conditions, a prominent band was observed at > 150 kDa for bibNAbs and monoclonal antibodies and corresponds to the purified human IgG control (right gel, Fig. 1c). Additional lower molecular weight bands were observed across all purified antibodies; including the human IgG control presumably indicating SDS detergent-sensitive heavy and light chain isoforms.

### Testing the functionality of the Fab moieties

**Fig. 2:**
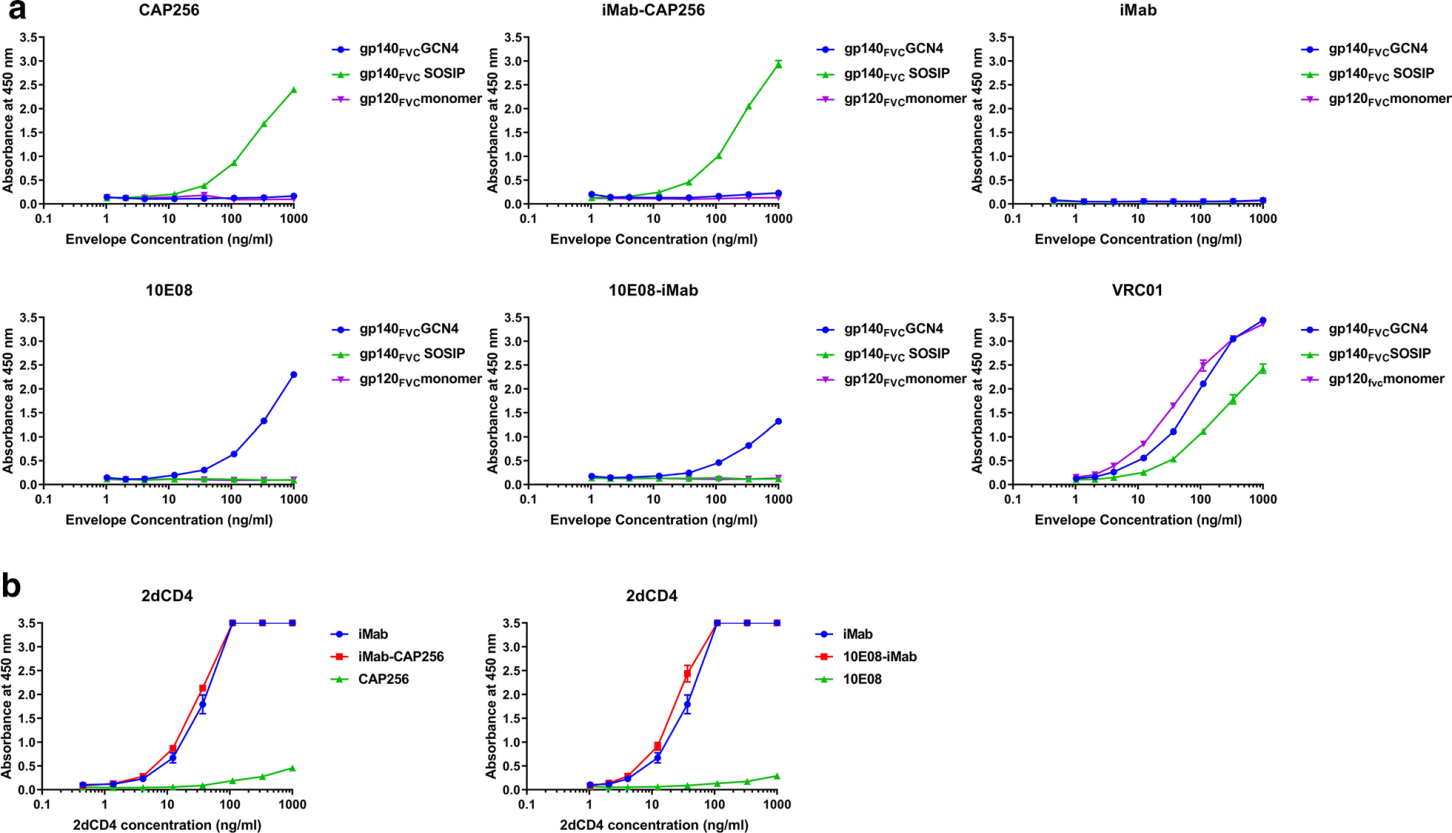
Binding characterisation of purified bispecific antibodies by ELISA. **a** HIV-1 Env binding of bNAbs and bibNAbs were assessed against different immobilized HIV-1 Env conformations (gp140_FVC_GN4, gp120_FVC_Monomer, gp140_FVC_SOSIP) and **b** the N-terminal two domain fragment of human CD4 (2dCD4) to assess their binding capabilities, specificity and bispecificity or lack thereof. The bNAbs and bibNAbs were tested at a concentration of 1 µg/ml except for VRC01, which was tested at 0.5 µg/ml. Secondary antibody concentration of 1 in 1000 was used for CAP256, iMab-CAP256, 10E08 and 10E08-iMab. For iMab and VRC01, a secondary antibody concentration of 1 in 2000 was used

Binding functionality of the HIV-1 Env targeting moieties (CAP256 or 10E08) and the host-directed anti-CD4 binding moiety, iMab was confirmed by ELISA (Fig. 2). Bispecific (iMab-CAP256 and 10E08-iMab) and parental (iMab, CAP256 and 10E08) monoclonal antibodies were tested against three different HIV-1 Env configurations based on the identical Env sequence (Consensus FVC sequence). These Env configurations included the gp120 monomeric, and the trimeric gp140_FVC_GCN4 and gp140_FVC_SOSIP configurations (Fig. 2a). As expected, CAP256, 10E08, iMab-CAP256 and 10E08-iMab did not bind to the gp120_FVC_ monomer, as this configuration lacks the gp41ecto domain containing the 10E08 epitope nor does the monomeric configuration recapitulate the quaternary epitope targeted by CAP256 (purple trace, Fig. 2a). CAP256 binding was evaluated using quaternary specific V1/V2 epitope stabilized on the soluble ‘native-like’ SOSIP trimer. Both the CAP256 parental and iMab-CAP256 bibNAb bound the gp140_FVC_SOSIP configuration confirming the functionality of these CAP256 moieties (green trace, Fig. 2a). The gp140_FVC_SOSIP trimer is truncated at position 664 and therefore lacks the 10E08 epitope. To confirm binding functionality of the 10E08 parental antibody and the 10E08 moiety on the 10E08-iMab bibNAb, antibody binding was therefore assessed using the gp140_FVC_GCN4 Env configuration. Both the parental 10E08 antibody and 10E08-iMab bibNAb bound the gp140_FVC_GCN4 configuration, confirming functionality of the 10E08 moiety on both 10E08 and 10E08-iMab (blue trace, Fig. 2a). The CD4 binding site-targeting bNAb, VRC01, was included as an appropriate positive control and confirms structural integrity of the three HIV-1 envelope configurations tested (Fig. 2a). No binding of the parental iMab antibody was detected against any of the HIV-1 Env configurations tested (Fig. 2a). Bacterially expressed, human 2dCD4 was used to confirm binding of the iMab parental antibody and iMab moiety of the bispecific antibody conformations (10E08-iMab and iMab-CAP256; Fig. 2b). The 10E08-iMab and iMab-CAP256 bispecific demonstrated similar levels of binding to immobilized 2dCD4 compared to the parental iMab antibody and confirms functionality of the iMab moiety in the context of the bispecific configuration (Fig. 2b).

### iMab-CAP256 displays enhanced neutralization potency over parental constituents

**Fig. 3:**
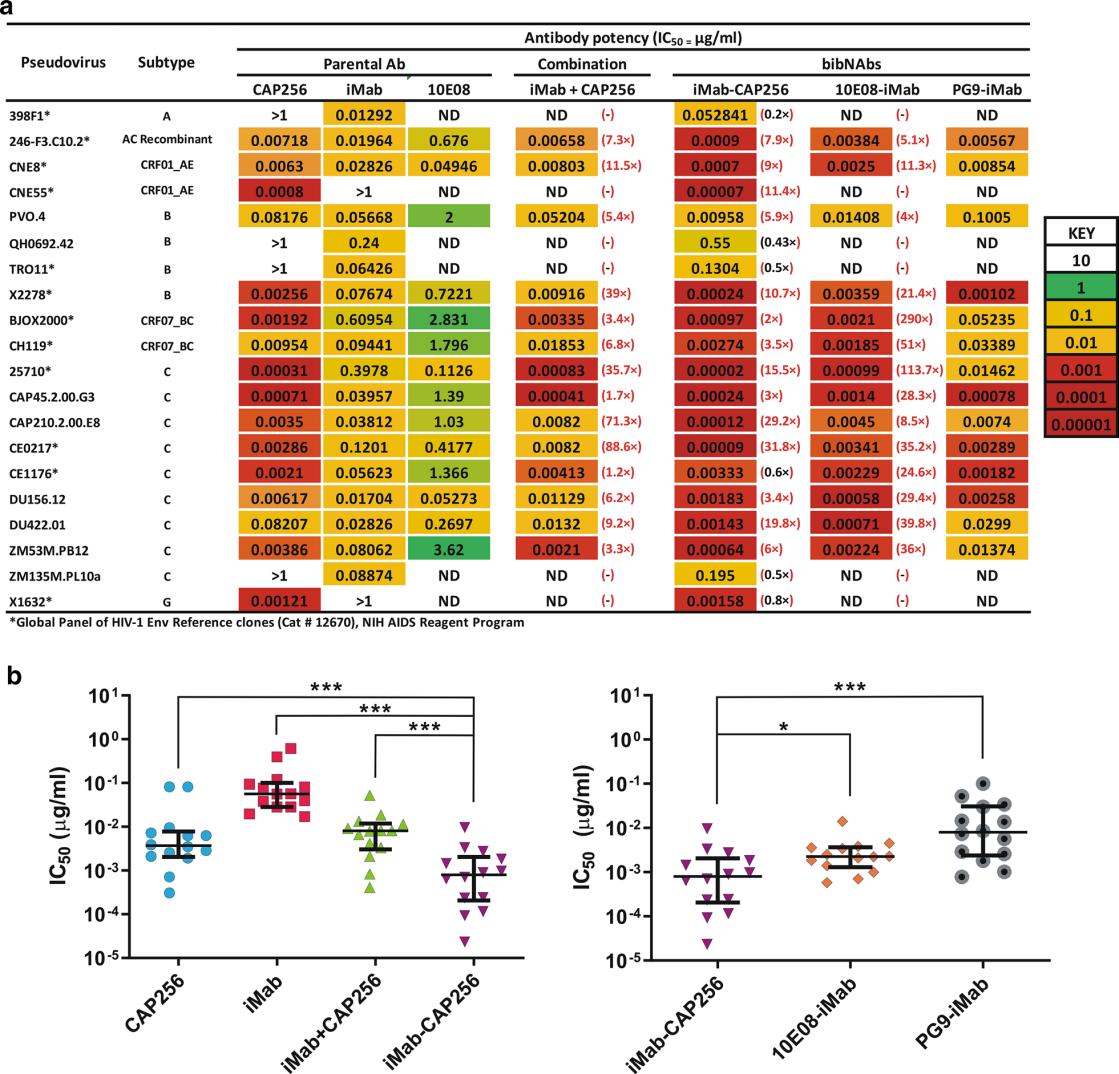
Differences in potency between bNAbs (individually and in combination) and bibNAbs against 20 geographically diverse HIV-1 pseudoviruses. **a** IC_50_ values were calculated for each parental bNAb, bNAb combination and bibNAb. Values in (red) indicate the fold increase in potency of the bibNAb iMab-CAP256 or 10E08-iMab compared to the more potent parental bNAb (either iMab, CAP256 or 10E08). Values (red) for the iMab + CAP256 combination show the fold increase in potency of the iMab-CAP256 relative to the combination. The smaller the IC_50_ or the darker the red, the more potent the Ab is. CAP256 resistant or iMab resistant viruses were included in the panel to test the neutralisation activity of both the CAP256 and iMab moieties in the bibNAb. **b** Scatter plots representing the median IC_50_ titres of parental antibodies (CAP256 and iMab), parental antibody combination (iMab + CAP256) and iMab-CAP256 bibNAb (left panel) or bibNAbs; iMab-CAP256, 10E08-iMab and PG9-iMab (right panel), against the CAP256/iMab dual sensitive pseudoviruses. Statistical differences in neutralization were determined using non-parametric *t*-test (Wilcoxon matched-pairs signed rank test) with *p < 0.05 and ***p < 0.001 represented. Errors bars indicate the interquartile ranges

To determine the potency of iMab-CAP256, a series of neutralisation assays were conducted against a panel of 20 geographically diverse, HIV-1 pseudoviruses (Fig. 3a). Selected pseudoviruses included single sensitive (n = 6; 398f1, CNE55, QH0692.42, TRO11, ZM135.PL10a and X1632) or dual sensitive (n = 14) strains to CAP256 and iMab parental antibodies. We initially compared iMab-CAP256 to its constituent parental antibodies, iMab and CAP256 (Fig. 3a). As expected, iMab-CAP256 showed improved breadth over the parental antibodies, neutralizing all 20 HIV-1 pseudoviruses tested, compared to 16/20 for CAP256 and 18/20 for iMab. Interestingly, iMab-CAP256 was consistently found to be more potent than the parental antibodies CAP256 (p = 0.0009) and iMab (p = 0.0001) against the dual sensitive pseudoviruses tested, with the exception of CE1176, as observed by the lower IC_50_ value (Fig. 3a, b left panel and Additional file 1: Figure S1). Overall, when the IC_50_ values for iMab-CAP256 were compared to the more potent parental antibody (iMab or CAP256) IC_50_ against each dual sensitive pseudovirus, the bibNAb demonstrated a 2 to 31.8 fold increase in potency (Fig. 3a).

In addition, iMab-CAP256 exhibited an 11.4 fold increase in potency compared to the parental CAP256 antibody against the iMab resistant CNE55 pseudovirus (Fig. 3a). This enhancement in potency and breadth of bibNAbs, including 10E08-iMab, has been demonstrated previously [47–50] and is reiterated here. The 10E08-iMab bispecific neutralized all viruses tested (n = 14) and showed a 4 to 290 fold enhancement in potency over the more potent parental antibody (iMab or 10E08) in each instance (Fig. 3a). Since the iMab-CAP256 only demonstrated potency enhancement against the dual sensitive pseudoviruses tested (with the exception of CE1176), subsequent screening of 10E08-iMab and PG9-iMab and the combination iMab + CAP256 focused on these. The relative fold-potency enhancement observed for 10E08-iMab compared to iMab-CAP256 over their respective parental antibodies, is primarily due to the lower potency of the 10E08 parental compared to CAP256 against the viruses tested. Neutralization potency of iMab-CAP256 was compared to the bispecific PG9-iMab [47] which shares antigenic targets on the HIV-1 Env (V1/V2 loop quaternary epitope—PG9 and CAP256) and host-CD4 receptor (iMab). iMab-CAP256 showed enhanced potency compared to PG9-iMAb for 13/14 viruses tested, with a median IC_50_ of 0.00079 compared to 0.0079 µg/ml (p = 0.0009, only CAP256/iMAb dual sensitive pseudoviruses were included in this analysis; Fig. 3a, b right panel and Additional file 2: Figure S2).

This observed potency enhancement of iMab-CAP256 over its parental antibody constituents suggests the presence of a synergistic/co-operative neutralization mechanism between these targeted epitopes. To elucidate whether this is a result of dual epitope targeting or simultaneous engagement of these two epitopes, the bibNAb, iMab-CAP256, was compared to a combination treatment of the parental antibodies (iMab + CAP256). This assay was conducted against the dual sensitive HIV-1 pseudoviruses using equivalent molar concentrations of antibody paratope binding regions (i.e. 1 µg/ml of iMab-CAP256 and 0.5 µg/ml final concentration of each parental antibody (total IgG concentration 1 µg/ml). iMab-CAP256 demonstrated enhanced potency (ranging from a 1.71 to 88.6 fold increase) over the parental combination treatment against all dual sensitive pseudoviruses tested (p = 0.0001, Fig. 3 and Additional file 3: Figure S3). The parental combination treatment demonstrated no improvement over the neutralization titres obtained for the more potent of the two parental antibodies, against the respective viruses tested (Fig. 3a and Additional file 3: Figure S3). This indicates that the bispecific combination of the CAP256 and iMab moieties reported here are crucial for the observed enhancement in potency.

## Discussion

In the absence of an effective, prophylactic HIV-1 vaccine, novel preventative strategies and therapeutics are needed to complement existing options. Technological advancements in the field of antigen specific, B-cell sorting have led to a dramatic increase in the discovery, isolation and characterisation of novel bNAbs from HIV-1 infected individuals [2–8, 55]. Clinical investigation of bNAb immunotherapy in HIV-1 infected patients and the prevention of HIV-1 in non-infected individuals are currently underway [36, 38, 39, 42, 79, 80]. BNAb combinations readily enhance the neutralization breadth and potency against a given HIV-1 pseudovirus panel [44, 45, 81], however these tend to be additive in nature [44]. Synergistic antibody combinations that result in increased potency have been reported e.g. PG9 + 10E8 [44], yet the improvements are limited and seldom exceed a twofold enhancement over the more potent, parental mAb in the combination. In contrast, bi/tribNAb configurations offer several advantages: their “single” molecule conformation supports manufacturing and regulatory registration convenience [53], while more importantly, their enhanced avidity may result in increased breadth and superior potency enhancement against HIV-1 [49, 50, 52–54].

We describe the engineering of a novel bispecific antibody comprising of the host-directed, anti-CD4 antibody iMab and HIV-1, V1/V2 targeting bNAb CAP256-VRC26.25 (CAP256). This bibNAb, iMab-CAP256, was designed using the CrossMab^CH1-CL^ [73] and knob-in-a-hole [50] mutations (Fig. 1a). Binding ELISA’s confirmed functionality of the individual Fab moieties and while this data is unable to confirm bispecificity in the true sense, i.e. simultaneous engagement of two differing antigenic targets, it clearly demonstrates binding functionality of the respective Fab moieties within this bibNAbs configuration (Fig. 2).

Despite the limited number of pseudoviruses tested, the neutralization breadth of the iMab-CAP256 was shown to be improved over the parental antibodies, neutralizing all viral strains tested (20/20) including single resistant strains (Fig. 3a). Neutralizing potency of the iMab-CAP256 bibNAb was also improved relative to the individual parental antibodies and the parental antibody combination, against all dual sensitive viral isolates tested except for CE1176 (Fig. 3a). Against the single resistant strains, iMab-CAP256 exhibited similar IC_50_ values to the parental antibody that demonstrated activity, except for CNE55, where iMab-CAP256 exhibited enhanced potency (Fig. 3a and Additional file 1: Figure S1).

Potency enhancement is highly desirable and a common feature shared amongst bibNAbs and tribNAbs reported in the literature [47, 49, 50, 52–54, 82]. This enhancement is attributed to improved avidity that allows for simultaneous epitope engagement [47, 49, 54], or intra-trimeric crosslinking [53, 82]. Interestingly, this enhancement in potency appears to be more pronounced when combining an Env and host-cell receptor targeting strategy [47, 49, 54]. The proposed mechanism is that anchoring of the bibNAbs to the cell surface via the iMab moiety effectively concentrates and allows for correct spatial positioning of the corresponding anti-Env paratope within the virus–host cell synapse, thereby enhancing the potency [47, 49].

Our iMab-CAP256 bibNAb clearly demonstrates enhanced potency that could not be reproduced by the parental combination (CAP256 + iMab) (Fig. 3 and Additional file 3: Figure S3). This suggests dual engagement of the targeted epitopes (CD4 and quaternary V1/V2 apical loop epitopes) and is in agreement with previously published findings that incorporate iMab into bibNAb configurations and reported potency enhancement [47, 49]. Closer inspection of the CNE55 neutralization curve (Additional file 1: Figure S1) indicates that although iMab did not achieve 50% inhibition over the concentrations tested, retention of iMab sensitivity, albeit to a lower degree, still facilitated the enhancement of neutralizing potency of the iMab-CAP256 bibNAb over the parental antibodies (iMab or CAP256).

IMab-CAP256 compared favourably to the PG9-iMab bibNAb against 15/16 of our selected pseudovirus isolate panel, with an approximate tenfold greater median IC_50_ (µg/ml) potency (Fig. 3b right panel). This was not unexpected given that the CAP256 parental mAb is more potent than PG9 and indeed the most potent within the V1/V2 glycan dependent class isolated to date [45, 55]. Although iMab-CAP256 and PG9-iMab share antigenic targets these bibNAbs configurations are structurally dissimilar. PG9-iMab incorporates the PG9 moiety as a single chain variable fragment via a 15-amino acid, glycine-serine linker peptide to the N-terminus of the iMab heavy chain [47]. This structural configuration provides greater avidity potential and enhanced flexibility over the CrossMab/knob-in-a-hole configuration. Whether further potency refinement of iMab-CAP256 may be achieved through structural optimization remains to be explored. Similarly, Haung et al. [49], reported on the PGT145-iMab bibNAb, CrossMab/knob-in-a-hole configuration which also shares antigenic targets with iMab-CAP256. Neutralization coverage of this bibNAb increased to above 95% yet moderate potency enhancement (< 10 fold enhancement) was observed compared to parental antibodies. Further work was not pursued on the PGT145-iMab bibNAb given the superior performance of 10E08-iMab reported in the same study [49]. However, their findings corroborate ours; showing potency enhancement was achieved using this epitope targeting combination, in the CrossMab/knob-in-a-hole configuration.

10E08-iMab has been described as the most potent and broadly neutralizing bibNAb characterised to date [49, 81]. It is unlikely iMab-CAP256 will exhibit broader neutralization coverage than 10E08-iMab, or PG9-iMab bibNAbs for that matter, given the reduced coverage of the parental CAP256 (57% of global isolates) in relation to 10E08 (98%) and PG9 (80%) [2, 8, 55]. However, the potency achieved by iMab-CAP256 may be comparable to, or outperform 10E08-iMab against certain HIV-1 subtypes. We observed a slightly lower, yet significant, difference in median IC_50_ values obtained for iMab-CAP256 compared to 10E08-iMab, against the pseudoviruses tested (Fig. 3b right panel). In addition, we present preliminary evidence of complementarity between the iMab-CAP256 and 10E08-iMab bibNAbs, as it was noted that the combined results neutralized 11/16 viral isolates (dual sensitive pseudoviruses only) at an IC_50_ value of < 0.001 µg/ml (Fig. 3a).

Ultimately, iMab-CAP256 will need to be evaluated against an extended HIV-1 pseudovirus panel, to validate the enhanced breath, potency and possible complementarity with 10E08-iMab. Of particular interest would be the inhibitory activity against HIV-1 subtype C viral isolates, given the worldwide predominance of this subtype and its specific affliction in the Sub-Saharan Africa region. In their follow up study, Wagh and colleague’s evaluated an updated bNAb panel that included the newly characterized bNAb, N6 [4] and two bibNAbs, 3BNC117-PGT135 [82] and 10E08_v2.0_-iMAb [49], against an expanded pseudovirus panel that included subtype A, C and D [81]. Dual antibody combinations comprising CAP256-VRC26.25 paired with N6 or 3BNC117 were shown to be the best-in-class against subtype C and D viral isolates, respectively. However, it should be noted that all dual bNAb combinations were surpassed by the single bibNAb, 10E8-iMAb across subtype A, C and D [81]. Once again, this study highlighted the superior advantage that may be attained through bi- and multi-specific antibody engineering compared to combination therapy. Indeed, further advantage was gained when the bibNAb, 10E08-iMab was used in combination with single bNAb N6 [81].

In conclusion, our results confirm the superiority of bibNAbs as compared to single bNAbs to neutralize HIV-1 in vitro. Moreover, incorporating CAP256.VRC26.235 into proven, multi-specific antibody configurations through class replacement (e.g. N6/PGDM1400-10E8v4 [52]) could provide further benefit. Thus, the iMab-CAP256 bibNAb configuration, and potential derivatives described here provides a unique and exciting opportunity for clinical development, given its improved neutralization breadth and potency.

## Supplementary information

**Additional file 1: Figure S1.** Breadth and potency of iMab-CAP256 in comparison to the parental bNAbs ibalizumab (iMab) and CAP256.VRC26.25 (CAP256). iMab-CAP256 shows greater potency than both the parental bNAbs for 13 of the 14 dual sensitive pseudoviruses tested as shown by the left shift of the bibNAb line on the graphs (median IC_50_ in µg/ml: iMab-CAP256 0.00079, iMab 0.056 and CAP256 0.0037). Against the six single resistant pseudoviruses (CAP256 resistant: 398F1, QH0692.4, TRO11 and ZM135.PL10a; iMab resistant: CNE55 and X1632), iMab-CAP256 has a potency similar to that of the active parental bNAb except for CNE55 were iMab-CAP256 exhibits enhanced potency. Unlike the parental bNAbs iMab and CAP256, iMab-CAP256 neutralised all 20 pseudoviruses tested thus demonstrating improved breath. All three Abs were tested at a maximum concentration of 1 µg/ml.

**Additional file 2: Figure S2.** Potency of iMab-CAP256 in comparison to 10E08-iMab and PG9-iMab. iMab-CAP256 shows on average, a potency on average 10× greater than PG9-iMab across all the 14 dual sensitive pseudoviruses tested except CE1176 (median IC_50_ for the 14 dual sensitive pseudoviruses: iMab-CAP256 0.00079, PG9-iMab 0.0079 µg/ml). Compared to 10E08-iMab, iMab-CAP256 is more potent against 11 of the 14 dual sensitive pseudoviruses tested (median IC_50_ for the 14 dual sensitive pseudoviruses in iMab-CAP256 is 0.00079 µg/ml, 10E08-iMab is 0.00226 µg/ml). iMab-CAP256 was tested at a maximum concentration of 1 µg/ml whilst PG9-iMab and 10E08-iMab were tested at 4 µg/ml.

**Additional file 3: Figure S3.** Potency of iMab-CAP256 in comparison to the parental bNAb combination (iMab+CAP256). BibNAb iMab-CAP256 shows a potency on average 10× greater than that of the parental combination iMab+CAP256 for all fourteen dual sensitive pseudoviruses tested (median IC_50_ in µg/ml: iMab-CAP256 0.00079, iMab+CAP256 0.0081). iMab-CAP256 was tested at 1 µg/ml and the bNAb combination was tested at 0.5 µg/ml of each parental Ab to achieve a total concentration of 1 µg/ml.

## Data Availability

The datasets used and/or analysed during the current study are available from the corresponding author on request.

## Abbreviations

ART: antiretroviral therapy
bNAbs: broadly neutralising antibodies
bibNAbs: bispecific bNAbs
CAP256: CAP256.VRC26.25
Env: HIV-1 envelope glycoprotein
Fab: fragment antigen-binding
FDA: Food and Drug Administration
HEK293T cells: Human embryonic kidney 293 T antigen cells
IC_50_: 50% inhibitory concentration
iMab: ibalizumab
iMab-CAP256: ibalizumab-CAP256.VRC26.25
PG9-iMab: PG9-ibalizumab
pMin: mAb expression vector
SDS-PAGE: sodium dodecyl sulphate polyacrylamide gel electrophoresis
SEC: size exclusion chromatography
TCID_50_: 50% Tissue Culture Infectious Dose
tribNAbs: trispecific bNAbs
